# TRIM14 regulates cell proliferation and invasion in osteosarcoma via promotion of the AKT signaling pathway

**DOI:** 10.1038/srep42411

**Published:** 2017-02-16

**Authors:** Guoxing Xu, Yongfei Guo, Dabo Xu, Yi Wang, Yafeng Shen, Feifei Wang, Yuanyuan Lv, Fanglong Song, Dawei Jiang, Yinquan Zhang, Yi Lou, Yake Meng, Yongji Yang, Yifan Kang

**Affiliations:** 1 Department of Biophysics, Second Military Medical University, No. 800 Xiangyin Road, 200433, Shanghai, People’s Republic of China; 2 Department of Orthopedics, ChangZheng Hospital, Second Military Medical University, No. 415 Fengyang Road, 200003, Shanghai, People’s Republic of China; 3 Department of Orthopedics, Third Affiliated Hospital, Second Military Medical University, No. 700 Moyu North Road, 201805, Shanghai, People’s Republic of China

## Abstract

Recent studies have shown that some members of the tripartite motif-containing protein (TRIM) family serve as important regulators of tumorigenesis. However, the biological role of TRIM14 in osteosarcoma remains to be established. In this study, we showed that TRIM14 is upregulated in human osteosarcoma specimens and cell lines, and correlated with osteosarcoma progression and shorter patient survival times. Functional studies demonstrated that overexpression of TRIM14 enhances osteosarcoma cell proliferation, clone formation, cell cycle procession, migration and invasion *in vitro* and promotes tumor growth *in vivo*, and conversely, its silencing has the opposite effects. Furthermore, TRIM14 overexpression induced activation of the AKT pathway. Inhibition of AKT expression reversed the TRIM14-mediated promotory effects on cell growth and mobility, in addition to TRIM14-induced epithelial-to-mesenchymal transition (EMT) and cyclin D1 upregulation. Our findings collectively suggest that TRIM14 functions as an oncogene by upregulating the AKT signaling pathway in osteosarcoma cells, supporting its potential utility as a therapeutic target for this disease.

Osteosarcoma is the most common malignant bone tumor type prevalent in children and young adults, and usually occurs in the metaphysis of long bones^1^,^2^. In the population aged 20 to 59 the incidence is significantly lower than 4/million/year^3^. Despite intensive application of combined multiagent chemotherapy and surgery, the 5-year survival rate of osteosarcoma patients with lung metastasis remains low, at 10–20%^4^,^5^. Therefore, effective identification and development of new targets for the diagnosis, treatment and prognosis of osteosarcoma remains an urgent medical requirement.

Tripartite motif-containing 14 (TRIM14), located at chromosome 9q22, is a member of the TRIM family^6^. TRIM proteins contain a series of conserved domains, including a RING (R) finger domain, one or two B-box motifs (B1 and B2), and a coiled coil (CC) region^7^. While some of the domains may be present or absent in different TRIM proteins (TRIM14 contains B1-CC domains but lacks the R domain), an order of R-B1-B2-CC is always maintained^8^. Accumulating evidence indicates that the TRIM family of proteins can act as either oncogenes or tumor suppressors to regulate various physiological processes, including cell proliferation, migration, invasion, apoptosis, cell cycle and differentiation^9^,^10^,^11^,^12^. Recent studies have reported upregulation of TRIM14 in tongue squamous cell carcinoma (TSCC) and non-small cell lung cancer (NSCLC) cells^13^,^14^. However, the expression levels and biological functions of TRIM14 in osteosarcoma progression remain largely unknown.

In the current study, we observed upregulation of TRIM14 in human osteosarcoma tissues and cell lines, which was strongly associated with aggressive characteristics and poor patient outcomes. Ectopic TRIM14 expression induced the growth, migration and invasion of osteosarcoma cells *in vitro* and promoted tumorigenesis in a mouse model, and conversely, knockdown of TRIM14 had the opposite effects. Clearly, TRIM14 plays an oncogenic role in osteosarcoma progression and may represent an effective therapeutic target for this disease.

## Materials and methods

### Cell culture

The human normal osteoblastic cell line, hFOB 1.19, and four human osteosarcoma cell lines, Saos-2, U2OS, MG-63, and HOS/MNNG, were purchased from the Cell Bank of the Chinese Academy of Sciences (Shanghai, China). Cell lines were cultured in Dulbecco’s modified Eagle’s medium (Gibco, Carlsbad, CA, USA) containing 10% fetal bovine serum (FBS), 1% penicillin and streptomycin (Invitrogen, Carlsbad, CA, USA) at 37 °C in a humidified atmosphere containing 5% CO_2_.

### Patient samples

In total, 45 primary osteosarcoma tissues and their matched adjacent normal bone tissues were obtained from Changhai Hospital (Shanghai, China). None of the patients had received preoperative treatment. All tissues were immediately frozen in liquid nitrogen after surgery and stored at −80 °C until use. Samples used were collected with informed consent from patients and approved by the ethics committee of Second Military Medical University, Shanghai, China. All the methods were carried out in accordance with the approved guidelines from Second Military Medical University.

### RNA extraction and quantitative real-time PCR (qRT-PCR)

Total RNA was extracted from primary osteosarcoma tissues and cells using TRIzol reagent (Invitrogen). cDNA synthesis was performed using the PrimeScript RT reagent Kit (TaKaRa, Dalian, China). qRT-PCR experiments were conducted utilizing the SYBR Green PCR Master Mix kit (Takara) on an ABI 7900 system (Applied Biosystems, Foster, CA, USA). mRNA expression of target genes was normalized to that of β-actin and calculated using the 2^−ΔΔct^ method. Primers used in qRT-PCR experiments were as follows: TRIM14: 5′-GCAGAAACTCAGCCAAGAA-3′ and 5′-CTTGACTCTGCATTAGCCT-3′, β-actin: 5′-GCGAGAAGATGACCCAGAT-3′ and 5′-AGGTAGTCAGGCAGTTCCC-3′.

### Lentivirus infection and transfection of siRNA

Lentiviral vectors expressing TRIM14, shRNA against TRIM14 or the respective controls were obtained from Hanbio (Shanghai, China). Saos-2 and HOS cell lines were infected with recombinant lentiviruses expressing TRIM14 or shTRIM14 in the presence of 8 μg/ml Polybrene (Sigma, St Louis, MO, USA). At 24 h after infection, virus-containing medium was removed and replaced with normal maintenance medium. After 48 h, successful transduction was confirmed via western blot. For transient transfection, small interfering RNA (siRNA) specific for AKT1 was purchased from Santa Cruz Biotechnology (Santa Cruz, CA, USA). Cells were transfected with AKT1 siRNA or a scrambled sequence using Lipofectamine 2000 reagent (Invitrogen) according to the manufacturer’s protocol. After 48 h, the efficiency of transfection was measured by western blot.

### Western blotting

Cells were lysed in RIPA (50 mM Tris-HCl, pH 7.5, 150 mM NaCl, 0.1% SDS, 0.5% deoxycholate, 1% NP-40) containing protease inhibitor cocktail and phophatase inhibitor cocktail. Protein concentrations were detected with the BCA protein assay (Pierce, Waltham, MA, USA), and 20 μg of each protein sample loaded onto 10% polyacrylamide gels for SDS-PAGE. Following transfer to PVDF membrane, blots were blocked in 5% milk in TBST and incubated with primary antibodies at 4 °C overnight. Primary antibodies against p-AKT, p-mTOR, p-p70S6K (Cell Signaling Technology, Danvers, MA, USA), β-actin, cyclin D1, TRIM14, AKT (Proteintech Group, Wuhan, Hubei, China), Vimentin and E-cadherin (Santa Cruz Biotechnology) were employed for blotting. After incubation with HRP-conjugated secondary antibody, bands were visualized with the ECL detection system (Millipore, Billerica, MA, USA). Bands were scanned and analysed with ImageJ (National Institutes of Health, Bethesda, MD, USA). β-Actin served as a loading control.

### Immunohistochemistry (IHC) analysis and scoring

Paraffin-embedded osteosarcoma tissues were deparaffinized, rehydrated, and subjected to a heat-induced epitope retrieval in 0.01 M sodium citrate (pH 6.0)^15^. Endogenous peroxidase activity was blocked with 0.3% hydrogen peroxide for 30 min. Sections were blocked with 10% goat serum in PBS for 30 min, followed by incubation with TRIM14 antibody at 4 °C overnight. After three washes with PBS, sections were incubated for 30 min each with biotin-labeled secondary antibody, and subsequently, streptavidin-peroxidase (Dako Diagnostics, Carpinteria, CA, USA). Sections were developed using 3, 3′-diaminobenzidine (DAB) substrate and counterstained with hematoxylin. Slides were dehydrated following a standard procedure and sealed with coverslips.

Immunohistochemical scores were assessed by two independent pathologists who had no knowledge of patient characteristics. Scores were assigned as intensity and percentage of positively stained tumor cells. The intensity of staining was divided into four grades: no staining (0), light brown (1), brown (2), and dark brown (3). Osteosarcoma samples were classified as positive in cases where TRIM14 staining intensity was >1 in >20% tumor cells.

### Cell proliferation assay

Cells were plated in 96-well plates at a density of 2 × 10^3^ cells per well and cultured for 24, 48, 72 and 96 h after transfection. MTT (10 μL of 5 mg/mL; Sigma) was added to each well, followed by incubation for 4 h. After the incubation step, the medium was discarded and 150 μL dimethyl sulfoxide added to solubilize formazan crystals. Absorbance at 490 nm was measured on a microplate reader.

### Colony formation assay

Cells plated onto six-well plates (500 cells per well) were cultured for 10 days. Colonies were fixed for 10 min with 10% formaldehyde, stained for 10 min with 1.0% crystal violet, counted and photographed. Each sample was tested in triplicate.

### Flow cytometry assay

Stably transfected cells were harvested at 72 h after transfection when confluence reached 80–95%, washed three times with PBS, and fixed with 70% ice-cold ethanol for at least 30 min. Cells were incubated with bovine pancreatic RNAase (2 μg/ml, Sigma) for 30 min at 37 °C, followed by propidium iodide solution (10 μg/ml, Invitrogen) for 30 min at room temperature. Cell cycle distributions were assessed using FACSCalibur (BD Biosciences, Bedford, MA, USA).

### Transwell migration and invasion assays

Transfected cells were serum-starved for 6 h. Cells (2.5 × 10^4^) in serum-free medium were seeded into the upper chamber of transwell filter chambers pre-coated with or without Matrigel (BD Biosciences) and medium supplemented with 10% FBS placed in the lower chamber. After 48 h of incubation at 37 °C, cells in the upper chamber were removed with a cotton swab. Cells that migrated and invaded the lower chamber were fixed in 4% formalin, stained with 0.1% crystal violet, imaged and counted.

### Tumor xenograft model

All experiments involving animals were performed in accordance with the National Institute of Health Guide for the Care and Use of Laboratory Animals, with the approval of the Scientific Investigation Board of Second Military Medical University, Shanghai, China. Cells (1 × 10^6^) infected with TRIM14, shRNA against TRIM14 or control lentivirus were injected subcutaneously into 4-week-old male BALB/C nude mice. Tumor volumes (V) were measured using the formula: V (mm^3^) = 0.5 × length × width^2^, and recorded every 5 days after inoculation. After 30 days, all tumor grafts were collected, photographed, fixed and embedded.

### Statistical analysis

All values were presented as means ± SD. SPSS 17.0 software was used for statistical analysis. Significant differences between the two groups were determined using the two-tailed Student’s *t*-test. Survival curves were plotted using Kaplan-Meier analyses and compared with the log-rank test. *P* values < 0.05 were considered statistically significant.

## Results

### TRIM14 is highly expressed in human osteosarcoma tissues and cell lines

**Figure 1:**
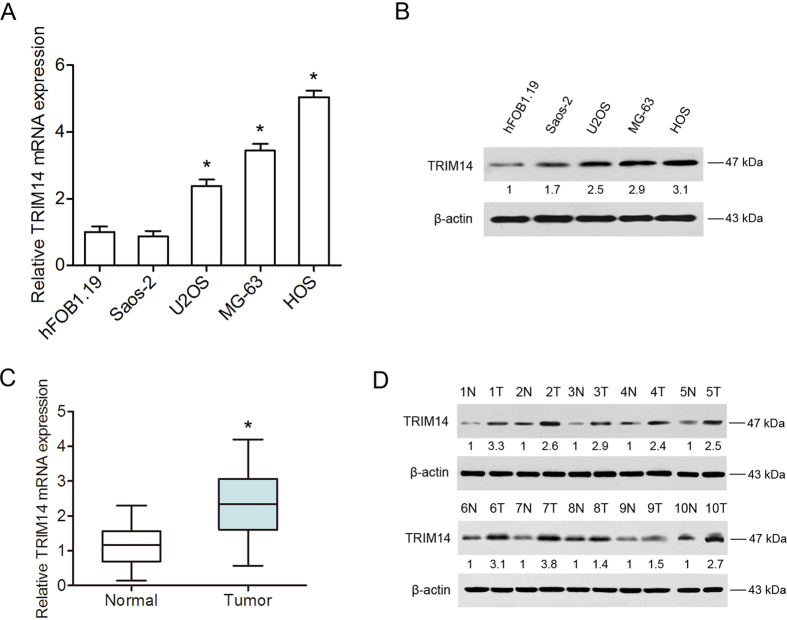
TRIM14 is highly expressed in human osteosarcoma tissues and cell lines. (**A**) TRIM14 mRNA and protein expression in human osteosarcoma cell lines and the normal osteoblastic cell line, hFOB 1.19, detected using qRT-PCR and western blot (**B**), respectively. The numbers below the blots show densitometric values that are corrected for loading. (**C**) TRIM14 mRNA expression in 45 osteosarcoma specimens and adjacent normal bone tissues. (**D**) Western blot analysis of TRIM14 protein expression. *P < 0.05.

Initially, TRIM14 mRNA and protein expression patterns were examined in four osteosarcoma cell lines (Saos-2, U2OS, MG-63, HOS) and the human normal osteoblastic cell line, hFOB 1.19, using qRT-PCR and western blot analyses. As shown in Fig. 1A and B, highest expression of TRIM14 was observed in HOS cells and lowest expression in Saos-2 and hFOB 1.19 cells. Next, we detected TRIM14 expression in human primary osteosarcoma tumors and adjacent normal bone tissues (Fig. 1C and D). The mRNA and protein levels of TRIM14 were significantly increased in osteosarcoma tissues, compared to their adjacent non-tumor tissues. The results collectively demonstrate that TRIM14 is overexpressed in human osteosarcoma tissues and cell lines.

### TRIM14 is correlated with progression and poor prognosis in human osteosarcoma

**Figure 2:**
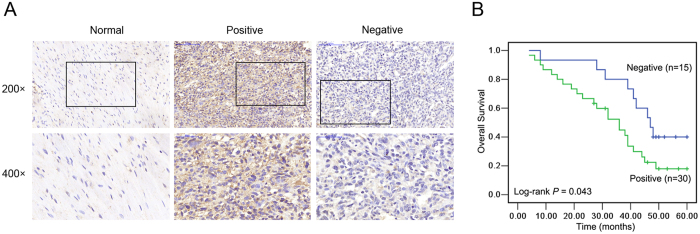
TRIM14 is correlated with poor prognosis in human osteosarcoma. (**A**) Representative images of TRIM14 expression in osteosarcoma specimens (middle and right) and normal bone tissues (left) examined by IHC. (**B**) Influence of TRIM14 expression patterns on overall survival estimated with Kaplan-Meier analyses.

We additionally examined protein expression of TRIM14 using immunohistochemistry (IHC) in 45 paraffin-embedded osteosarcoma tissues. Compared to the corresponding adjacent normal bone tissues, protein levels of TRIM14 were higher in osteosarcoma tissues (Fig. 2A). Among the osteosarcoma tissues, 67% (30/45) cases were classified as TRIM14-positive, whereas 33% (15/45) stained negative for TRIM14. The correlations between TRIM14 expression and clinicopathologic features of osteosarcoma were further analyzed. As shown in Table 1, expression of TRIM14 was significantly associated with higher tumor stage (*P* = 0.038) and histologic grade (*P* = 0.012). Kaplan-Meier survival analysis revealed that TRIM14 protein levels are linked with poor prognosis in osteosarcoma patients. Patients with positive TRIM14 expression displayed significantly poorer overall survival than those with negative TRIM14 expression (Fig. 2B). These findings indicate that increased expression of TRIM14 is associated with osteosarcoma progression as well as shorter patient survival.

### Overexpression of TRIM14 promotes osteosarcoma cell proliferation, migration and invasion

**Figure 3:**
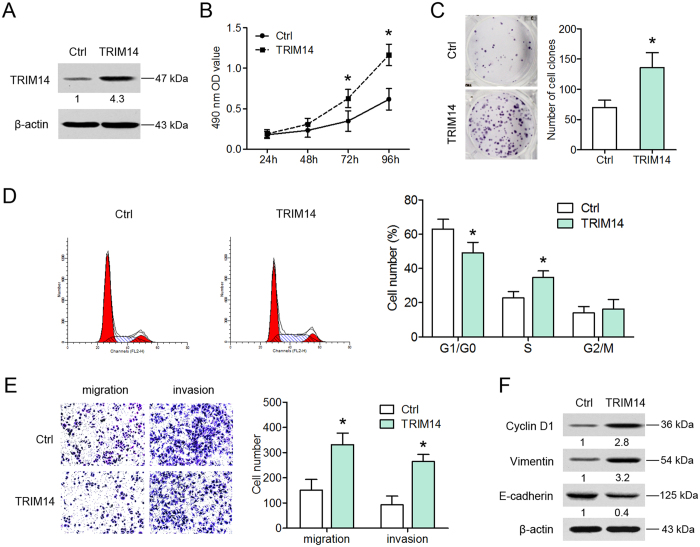
Overexpression of TRIM14 promotes osteosarcoma cell proliferation, migration and invasion. (**A**) Saos-2 cells were infected with TRIM14-expressing (TRIM14) or control lentivirus (Ctrl), and TRIM14 protein levels assessed using western blot. (**B**) Cell viability was measured on the indicated days using the MTT assay. (**C**) Representative micrographs (left) and quantification (right) of osteosarcoma cell colonies detected with the colony formation assay. (**D**) Flow cytometry analysis of cell cycle progression in osteosarcoma cells. (**E**) Cell migration and invasion were determined via Transwell assays. Representative images (left) and quantification (right) of the migration and invasion assays in the indicated cells. (**F**) Western blot analysis of cyclin D1, vimentin and E-cadherin protein expression. *P < 0.05.

To investigate the biological function of TRIM14 in pathogenesis of osteosarcoma, stable TRIM14-expressing Saos-2 cells were established (Fig. 3A). Data from the cell viability assay showed that TRIM14 overexpression significantly promotes the proliferation of Saos-2, compared with control cells (Fig. 3B). Furthermore, more colonies were formed by stable TRIM14-expressing Saos-2 cells than control cells (Fig. 3C). Consistent with these findings, enforced expression of TRIM14 led to a decreased percentage of cells in the G1 phase along with an increase in S-phase cells (Fig. 3D). To determine the effects of TRIM14 on the migration and invasion abilities of osteosarcoma cells, we performed Transwell assays. Notably, ectopic expression of TRIM14 enhanced the number of migrating and invading osteosarcoma cells (Fig. 3E). Next, western blotting was performed to examine the expression levels of cyclin D1 (a key cell cycle regulator) and EMT markers (the mesenchymal marker, vimentin, and the epithelial marker, E-cadherin). As shown in Fig. 3F, TRIM14-expressing cells displayed increased expression of cyclin D1 and vimentin and simultaneous downregulation of E-cadherin. Similar patterns of cell proliferation, migration and invasion were found in TRIM14-overexpressing HOS cells (Supplementary Fig. 1). Based on these findings, we propose that TRIM14 accelerates osteosarcoma cell growth, migration and invasion.

### Inhibition of TRIM14 suppresses the proliferation, migration and invasion of osteosarcoma cells

**Figure 4:**
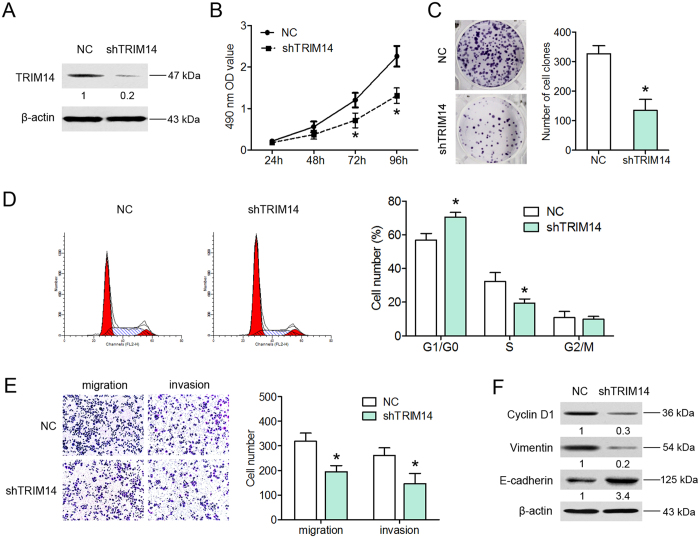
Inhibition of TRIM14 suppresses the proliferation, migration and invasion of osteosarcoma cells. (**A**) Western blot of TRIM14 protein expression in HOS cells infected with shRNA against TRIM14 (shTRIM14) or negative control lentivirus (NC). Silencing of endogenous TRIM14 inhibited cell viability, clone formation, cell cycle, migration and invasion, as determined from the MTT (**B**), colony formation (**C**), flow cytometry (**D**), and Transwell migration and invasion assays (**E**). (**F**) Protein expression of cyclin D1, vimentin and E-cadherin. *P < 0.05.

To further validate the promotory effects of TRIM14 on osteosarcoma cell growth, and mobility, we suppressed TRIM14 expression in HOS cells using short hairpin RNAs (Fig. 4A). Depletion of endogenous TRIM14 led to a dramatic reduction in cell viability and colony number, compared to control cells (Fig. 4B and C). Moreover, following TRIM14 silencing, we detected a marked decrease in the percentage of S-phase and increased proportion of G1/G0 phase cells (Fig. 4D). TRIM14 knockdown suppressed the migration and invasion ability of osteosarcoma cells (Fig. 4E), and induced downregulation of cyclin D1 and vimentin and simultaneous upregulation of E-cadherin (Fig. 4F). Similarly, Saos-2 cells with TRIM14 depletion exhibited decreased proliferation, invasion and migration abilities compared to control cells (Supplementary Fig. 1). The collective results indicate that knockdown of TRIM14 inhibits osteosarcoma cell proliferation and mobility.

### TRIM14 regulates osteosarcoma tumor growth *in vivo*

**Figure 5:**
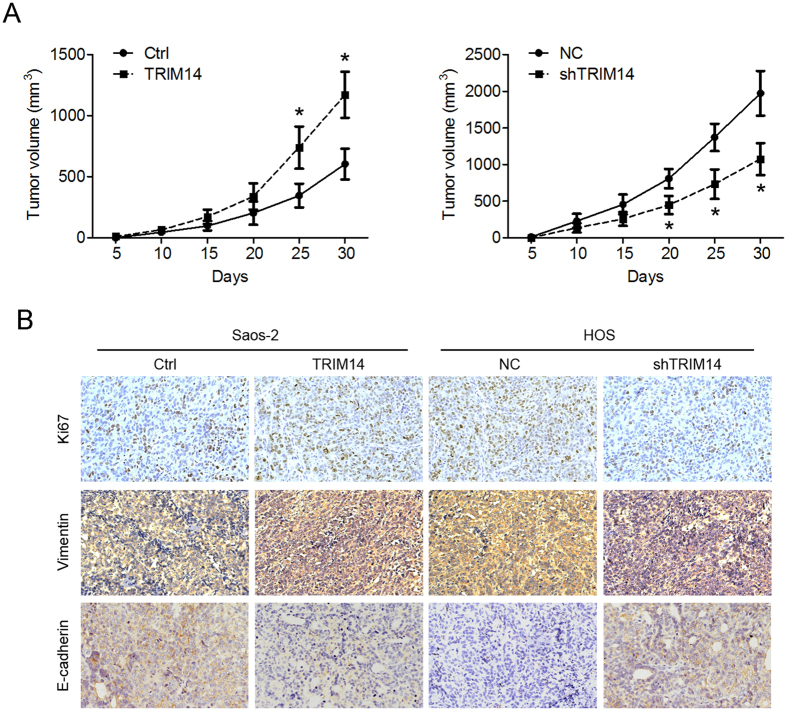
TRIM14 regulates osteosarcoma tumor growth *in vivo*. (**A**) Stable TRIM14-expressing Saos-2 or TRIM14-silenced HOS cells were injected subcutaneously into nude mice. Tumor volumes were measured, and presented as growth curves. (**B**) Tumor sections were subjected to IHC staining using Ki-67, vimentin or E-cadherin antibody. *P < 0.05.

We further investigated the effects of TRIM14 overexpression on osteosarcoma tumor growth *in vivo*. Stable TRIM14-expressing Saos-2 cells or TRIM14-silenced HOS cells were subcutaneously inoculated into nude mice. As indicated in Fig. 5A, tumors formed by TRIM14-expressing cells showed faster growth and greater sizes than those formed by control Saos-2 cells. Conversely, tumors formed in TRIM14-silenced cells grew slower with smaller sizes than those generated from the cell group expressing control shRNA. IHC analysis revealed a higher number of Ki-67-positive cells, higher vimentin expression and lower E-cadherin expression in TRIM14-expressing tumors and the opposite findings in TRIM14-silenced tumors (Fig. 5B). Our results clearly support an oncogenic role of TRIM14 in osteosarcoma *in vivo*.

### TRIM14 regulates cell proliferation and metastasis via the AKT signaling pathway

**Figure 6:**
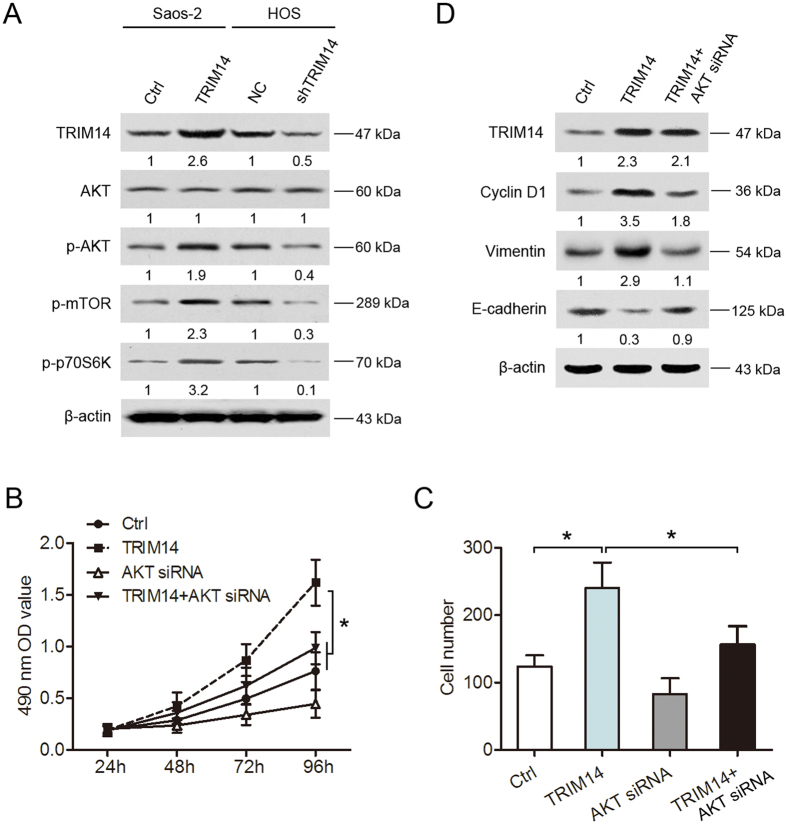
TRIM14 regulates osteosarcoma cell growth and invasion via activating the AKT signaling pathway. (**A**) Western blot analysis of the indicated proteins in cells. (**B**) Saos-2/TRIM14 cells were treated with or without AKT siRNA, for 48 h, and cell proliferation and invasion abilities determined with the MTT and Transwell invasion assays, respectively (**C**). (**D**) Western blot analysis of the indicated proteins after treatment with AKT siRNA. siRNA, small interfering RNA. *P < 0.05.

The AKT pathway plays a critical role in regulation of cell growth and metastasis^16^. In view of the finding that members of the TRIM protein family, such as TRIM29 and TRIM44, stimulate the AKT pathway in human cancer^17^,^18^, we investigated whether TRIM14 is associated with the AKT pathway in osteosarcoma. Notably, TRIM14 overexpression enhanced the levels of p-AKT, p-mTOR and p-P70S6K whereas its inhibition had the opposite effects (Fig. 6A). To ascertain whether the proliferation and metastasis of osteosarcoma cells induced by TRIM14 result from activation of the AKT pathway, we examined the growth and invasion abilities of TRIM14-expressing cells after suppression of AKT signaling using siRNA targeting AKT (Supplementary Fig. 2). As illustrated in Fig. 6B and C, suppression of AKT significantly blocked the promotory effects of TRIM14 on cell proliferation and invasion. Similarly, western blot analysis of cyclin D1 and EMT markers revealed that inhibition of AKT markedly diminished the effects of TRIM14 (i.e., the increase in cyclin D1 and vimentin and decrease in E-cadherin expression) in Saos-2 cells (Fig. 6D). The data collectively demonstrate that TRIM14 activity is mediated, at least in part, by the AKT pathway, which contributes to its promotory effects on osteosarcoma cell proliferation and invasion.

## Discussion

TRIM14 belongs to the TRIM protein family, which contains more than 70 members. Alterations in these proteins are linked to different pathological conditions, including developmental disorders, neurodegenerative diseases, viral infections and cancer^19^. Previous studies have reported increased levels of TRIM14 in many cell types, which are associated with regulation of diverse basic cellular functions^13^,^14^,^20^,^21^. For example, TRIM14 is significantly upregulated in A549 cells and accelerates the retinoic acid-inducible gene-I-like receptor-mediated innate immune response^14^,^21^. Additionally, TRIM14 has been shown to be markedly increased in TSCC cell lines and clinical tissues, and correlated with poor prognosis, promotion of cell growth *in vitro* and *in vivo*, angiogenesis and invasion, and enhanced resistance to cisplatin-induced apoptosis^13^. Consistent with earlier findings, our data showed high expression of TRIM14 in osteosarcoma cell lines and tissues in association with aggressive clinical features and unfavorable prognosis. Upregulation of TRIM14 promoted cell proliferation and tumorigenicity, migration and invasion, while inhibition of TRIM14 had the opposite effects.

To explore the mechanism underlying TRIM14-induced cell growth and metastasis in osteosarcoma cells, we examined its effects on AKT signaling, known to be activated by the TRIM protein family. The AKT signaling pathways represent a cascade of events involved in various physiological and pathological processes, including cell proliferation, migration, invasion, differentiation and apoptosis^22^,^23^,^24^,^25^,^26^. In colorectal cancer, inhibition of the AKT signaling pathway suppressed cell proliferation, clone formation *in vitro* and tumor growth *in vivo*^23^. Additionally, activation of AKT signaling enhanced gallbladder cancer cell proliferation, metastasis and tumor growth^24^. A recent study by Shen *et al*.^25^ showed that an increase in AKT signaling induces osteosarcoma cell proliferation, migration and tumor formation. Consistent with earlier findings, TRIM14 increased p-AKT, p-mTOR and p-P70S6K levels in osteosarcoma cells, and blocking AKT activity reversed the TRIM14-induced promotory effects on cell growth and metastasis in the current study.

Abnormal AKT kinase activation contributes to oncogenesis via affecting the regulation of multiple downstream molecules^27^,^28^. Cyclin D1 is an important cell cycle regulator downstream of the AKT pathway^29^. AKT activation is reported to increase cyclin D1 expression to regulate cell growth^30^. Activation of AKT also promotes epithelial-mesenchymal transition (EMT), an important step in tumor invasion and metastasis^31^. In our experiments, TRIM14 expression led to enhanced cyclin D1 and vimentin and reduced E-cadherin levels. Notably, inhibition of AKT expression by siRNA suppressed cyclin D1 and vimentin levels and increased E-cadherin expression in osteosarcoma cells. Our results collectively indicate that regulation of cyclin D1, vimentin and E-cadherin by TRIM14 results from alterations in AKT activity. Accordingly, we propose that TRIM14 promotes the proliferation, migration and invasion of osteosarcoma cells via upregulation of the AKT pathway.

In conclusion, TRIM14 is markedly overexpressed in osteosarcoma cell lines and clinical samples, and facilitates cell growth and mobility by modulating the AKT pathway. These findings aid in improving our understanding of osteosarcoma progression and support the potential utility of TRIM14 as an attractive therapeutic target for cancer.

## Additional Information

**How to cite this article**: Xu, G. *et al*. TRIM14 regulates cell proliferation and invasion in osteosarcoma via promotion of the AKT signaling pathway. *Sci. Rep.* **7**, 42411; doi: 10.1038/srep42411 (2017).

**Publisher's note:** Springer Nature remains neutral with regard to jurisdictional claims in published maps and institutional affiliations.

## Supplementary Material

Supplementary Data

## Figures and Tables

**Table 1:** Clinicopathologic variables and the expression status of TRIM14 in 45 osteosarcoma patients.

Clinical pathologic parameters		N	TRIM14 negative	TRIM14 positive	P-Value
Age (yr)	<30	30	12	18	0.315
	≥30	15	3	12	
Sex	Female	22	9	13	0.353
	Male	23	6	17	
Tumor size (cm)	≤8	21	10	11	0.112
	>8	24	5	19	
Stage	I–II	33	14	19	**0.038**
	III–IV	12	1	11	
Distant metastasis	Absence	35	10	25	0.263
	Presence	10	5	5	
Histological grade	Low (well and moderately differentiated)	6	5	1	**0.012**
	High (Poorly differentiated)	39	10	29	
Histological subtype	Osteoblastic	23	7	16	0.873
	Chondroblastic	8	3	5	
	Fibroblastic	4	2	2	
	Others	10	3	7	

## References

[b1] Cheng D. D. . MiR-542-5p is a negative prognostic factor and promotes osteosarcoma tumorigenesis by targeting HUWE1. Oncotarget 6, 42761–72 (2015). 26498360 10.18632/oncotarget.6199 PMC4767468

[b2] Wagle S. . DBC1/CCAR2 is involved in the stabilization of androgen receptor and the progression of osteosarcoma. Sci Rep 5, 13144 (2015). 26249023 10.1038/srep13144 PMC4642542

[b3] Han K. . MicroRNA profiling identifies MiR-195 suppresses osteosarcoma cell metastasis by targeting CCND1. Oncotarget 6, 8875–89 (2015). 25823925 10.18632/oncotarget.3560 PMC4496189

[b4] Bi Y., Jing Y. & Cao Y. Overexpression of miR-100 inhibits growth of osteosarcoma through FGFR3. Tumour Biol 36, 8405–11 (2015). 26018508 10.1007/s13277-015-3581-1

[b5] Wang W., Zhou X. & Wei M. MicroRNA-144 suppresses osteosarcoma growth and metastasis by targeting ROCK1 and ROCK2. Oncotarget 6, 10297–308 (2015). 25912304 10.18632/oncotarget.3305 PMC4496356

[b6] Meroni G. & Diez-Roux G. TRIM/RBCC, a novel class of ‘single protein RING finger’ E3 ubiquitin ligases. Bioessays 27, 1147–57 (2005). 16237670 10.1002/bies.20304

[b7] Reymond A. . The tripartite motif family identifies cell compartments. EMBO J 20, 2140–51 (2001). 11331580 10.1093/emboj/20.9.2140 PMC125245

[b8] Wang L. . Oncogenic function of ATDC in pancreatic cancer through Wnt pathway activation and beta-catenin stabilization. Cancer Cell 15, 207–19 (2009). 19249679 10.1016/j.ccr.2009.01.018 PMC2673547

[b9] Chen Y. . TRIM66 overexpresssion contributes to osteosarcoma carcinogenesis and indicates poor survival outcome. Oncotarget 6, 23708–19 (2015). 26247633 10.18632/oncotarget.4291 PMC4695146

[b10] Wang Y. . TRIM26 functions as a novel tumor suppressor of hepatocellular carcinoma and its downregulation contributes to worse prognosis. Biochem Biophys Res Commun 463, 458–65 (2015). 26043685 10.1016/j.bbrc.2015.05.117

[b11] Zhu Z. . TRIM25 blockade by RNA interference inhibited migration and invasion of gastric cancer cells through TGF-beta signaling. Sci Rep 6, 19070 (2016). 26754079 10.1038/srep19070 PMC4709557

[b12] Marshall G. M. . TRIM16 acts as a tumour suppressor by inhibitory effects on cytoplasmic vimentin and nuclear E2F1 in neuroblastoma cells. Oncogene 29, 6172–83 (2010). 20729920 10.1038/onc.2010.340 PMC3007621

[b13] Su X. . Overexpression of TRIM14 promotes tongue squamous cell carcinoma aggressiveness by activating the NF-kappaB signaling pathway. Oncotarget 7, 9939–50 (2016). 26799420 10.18632/oncotarget.6941 PMC4891094

[b14] Zhan W. . TRIM59 Promotes the Proliferation and Migration of Non-Small Cell Lung Cancer Cells by Upregulating Cell Cycle Related Proteins. PLoS One 10, e0142596 (2015). 26599082 10.1371/journal.pone.0142596 PMC4658198

[b15] Zhou Z. . TRIM59 is up-regulated in gastric tumors, promoting ubiquitination and degradation of p53. Gastroenterology 147, 1043–54 (2014). 25046164 10.1053/j.gastro.2014.07.021

[b16] Kong L. . Lamin A/C protein is overexpressed in tissue-invading prostate cancer and promotes prostate cancer cell growth, migration and invasion through the PI3K/AKT/PTEN pathway. Carcinogenesis 33, 751–9 (2012). 22301279 10.1093/carcin/bgs022

[b17] Zhou X. M. . Upregulated TRIM29 promotes proliferation and metastasis of nasopharyngeal carcinoma via PTEN/AKT/mTOR signal pathway. Oncotarget (2016). 10.18632/oncotarget.7215 PMC4924667 26872369

[b18] Xing Y. . TRIM44 promotes proliferation and metastasis in nonsmall cell lung cancer via mTOR signaling pathway. Oncotarget (2016). 10.18632/oncotarget.8586 PMC5058694 27058415

[b19] Hatakeyama S. TRIM proteins and cancer. Nat Rev Cancer 11, 792–804 (2011). 21979307 10.1038/nrc3139

[b20] Kimsa M. W. . Differential expression of tripartite motif-containing family in normal human dermal fibroblasts in response to porcine endogenous retrovirus infection. Folia Biol (Praha) 60, 144–51 (2014). 25056437 10.14712/fb2014060030144

[b21] Zhou Z. . TRIM14 is a mitochondrial adaptor that facilitates retinoic acid-inducible gene-I-like receptor-mediated innate immune response. Proc Natl Acad Sci USA 111, E245–54 (2014). 24379373 10.1073/pnas.1316941111 PMC3896185

[b22] Zhang J., Yu X. H., Yan Y. G., Wang C. & Wang W. J. PI3K/Akt signaling in osteosarcoma. Clin Chim Acta 444, 182–92 (2015). 25704303 10.1016/j.cca.2014.12.041

[b23] Zhou W. . The tumor-suppressor gene LZTS1 suppresses colorectal cancer proliferation through inhibition of the AKT-mTOR signaling pathway. Cancer Lett 360, 68–75 (2015). 25667121 10.1016/j.canlet.2015.02.004

[b24] Zhang Y. . A novel PI3K/AKT signaling axis mediates Nectin-4-induced gallbladder cancer cell proliferation, metastasis and tumor growth. Cancer Lett 375, 179–89 (2016). 26949052 10.1016/j.canlet.2016.02.049

[b25] Shen G. . MicroRNA-105 suppresses cell proliferation and inhibits PI3K/AKT signaling in human hepatocellular carcinoma. Carcinogenesis 35, 2748–55 (2014). 25280563 10.1093/carcin/bgu208

[b26] Daniele S. . Combined inhibition of AKT/mTOR and MDM2 enhances Glioblastoma Multiforme cell apoptosis and differentiation of cancer stem cells. Sci Rep 5, 9956 (2015). 25898313 10.1038/srep09956 PMC4404683

[b27] Cui Y. M. . FOXC2 promotes colorectal cancer proliferation through inhibition of FOXO3a and activation of MAPK and AKT signaling pathways. Cancer Lett 353, 87–94 (2014). 25069037 10.1016/j.canlet.2014.07.008

[b28] Chen J. S. . Sonic hedgehog signaling pathway induces cell migration and invasion through focal adhesion kinase/AKT signaling-mediated activation of matrix metalloproteinase (MMP)-2 and MMP-9 in liver cancer. Carcinogenesis 34, 10–9 (2013). 22948179 10.1093/carcin/bgs274

[b29] Chen J. . Gankyrin facilitates follicle-stimulating hormone-driven ovarian cancer cell proliferation through the PI3K/AKT/HIF-1alpha/cyclin D1 pathway. Oncogene (2015). 10.1038/onc.2015.316 26364616

[b30] Tong Z. T. . AIB1 predicts bladder cancer outcome and promotes bladder cancer cell proliferation through AKT and E2F1. Br J Cancer 108, 1470–9 (2013). 23511556 10.1038/bjc.2013.81 PMC3629431

[b31] Grille S. J. . The protein kinase Akt induces epithelial mesenchymal transition and promotes enhanced motility and invasiveness of squamous cell carcinoma lines. Cancer Res 63, 2172–8 (2003). 12727836

