# Supplementary material for: TRIM14 regulates cell proliferation and invasion in osteosarcoma via promotion of the AKT signaling pathway
Source: Sci Rep. 2017 Feb 16;7:42411. doi: 10.1038/srep42411 (PMC5311867; doi:10.1038/srep42411)

TRIM14 regulates cell proliferation and invasion in osteosarcoma via promotion of the AKT signaling pathway

Guoxing Xu[1,*], Yongfei Guo[2,*], Dabo Xu[3,*], Yi Wang[3], Yafeng Shen[1], Feifei Wang[1], Yuanyuan Lv[1], Fanglong Song[3], Dawei Jiang[3], Yinquan Zhang[3], Yi Lou[3], Yake Meng[2], Yongji Yang[1] & Yifan Kang[3]

[1]Department of Biophysics, Second Military Medical University, No. 800 Xiangyin Road, 200433, Shanghai, People's Republic of China.

[2]Department of Orthopedics, ChangZheng Hospital, Second Military Medical University, No. 415 Fengyang Road, 200003, Shanghai, People's Republic of China.

[3]Department of Orthopedics, Third Affiliated Hospital, Second Military Medical University, No. 700 Moyu North Road, 201805, Shanghai, People's Republic of China.

[*]These authors contributed equally to this work.

Correspondence and requests for materials should be addressed to Y.-J. Y. (email: yjyang22@163.com) or Y.-F. K. (yifankangch@sina.com)

Supplement Figure Legends

Supplementary Figure 1. TRIM14 promotes osteosarcoma cell proliferation, migration and invasion. (**A**) Western blot analysis of TRIM14 protein expression in HOS cells with TRIM14 overexpression or Saos-2 cells with TRIM14 knockdown. Overexpression of TRIM14 in HOS cells promoted cell proliferation (**B**), clone formation (**C**), cell cycle progression (**D**), migration and invasion (**E**), and induced EMT and cyclin D1 upregulation (**F**). Conversely, knockdown of TRIM14 in Saos-2 cells has the opposite effects. $^*P < 0.05$.

Supplementary Figure 2. Western blot analysis of AKT protein expression in Saos-2 cells transfected with scramble siRNA or AKT siRNA. AKT siRNA resulted in effective knockdown of AKT expression.

Supplementary Figure 1.

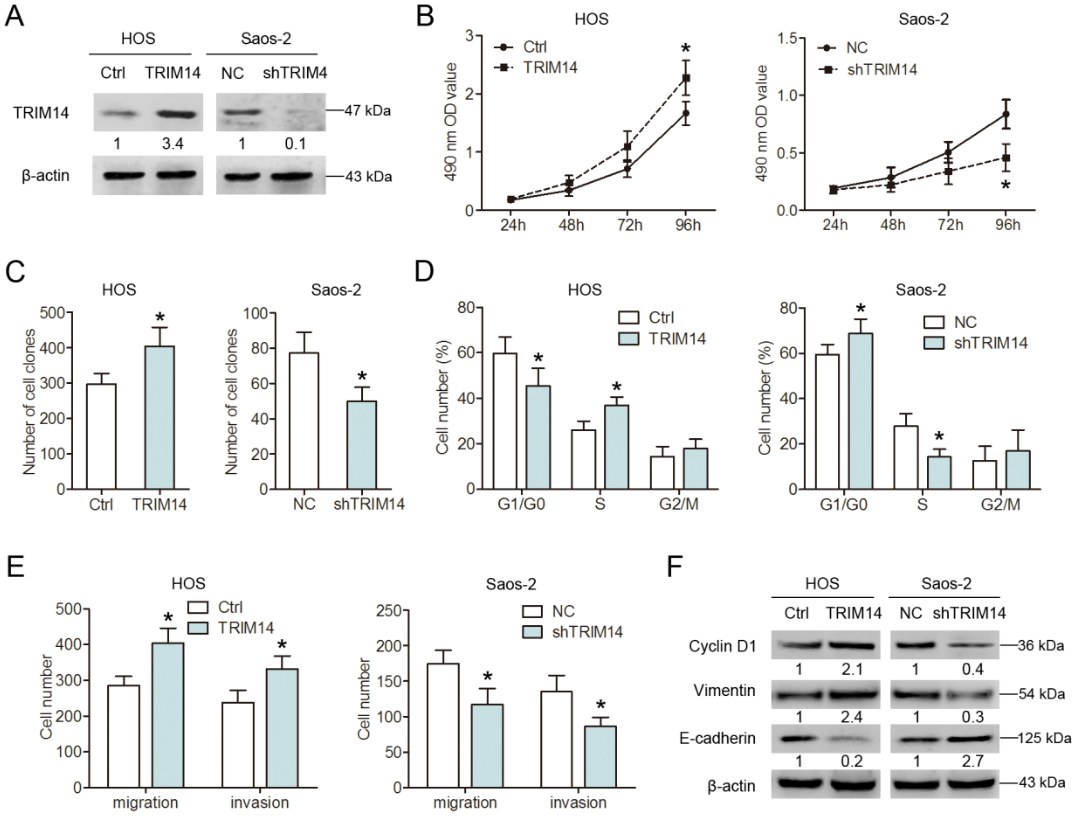

Supplementary Figure 2.

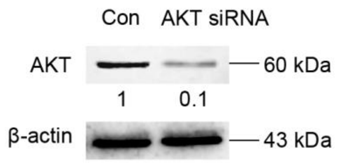

Figure 1B

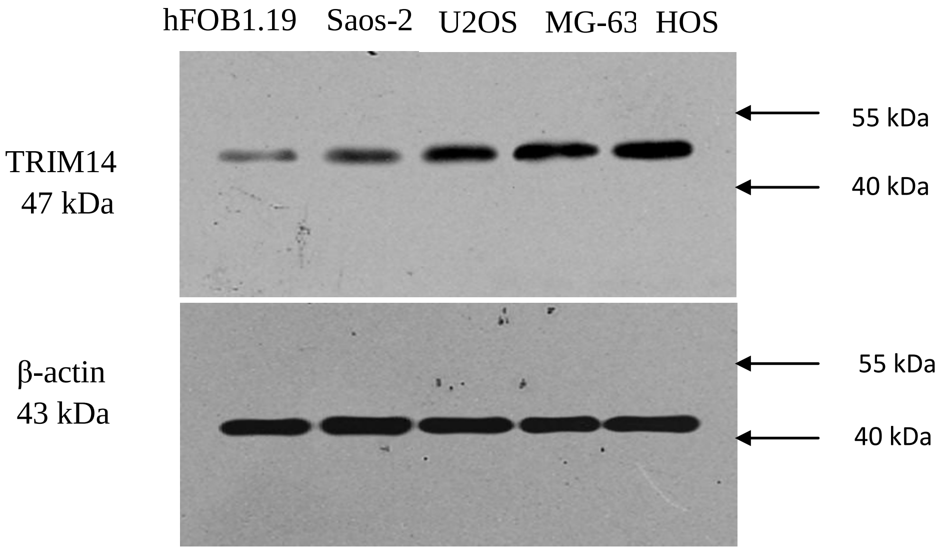
hFOB1.19
Saos-2
U2OS
MG-63
HOS
TRIM14
47 kDa
55 kDa
40 kDa
β-actin
43 kDa
55 kDa
40 kDa

Figure 1D

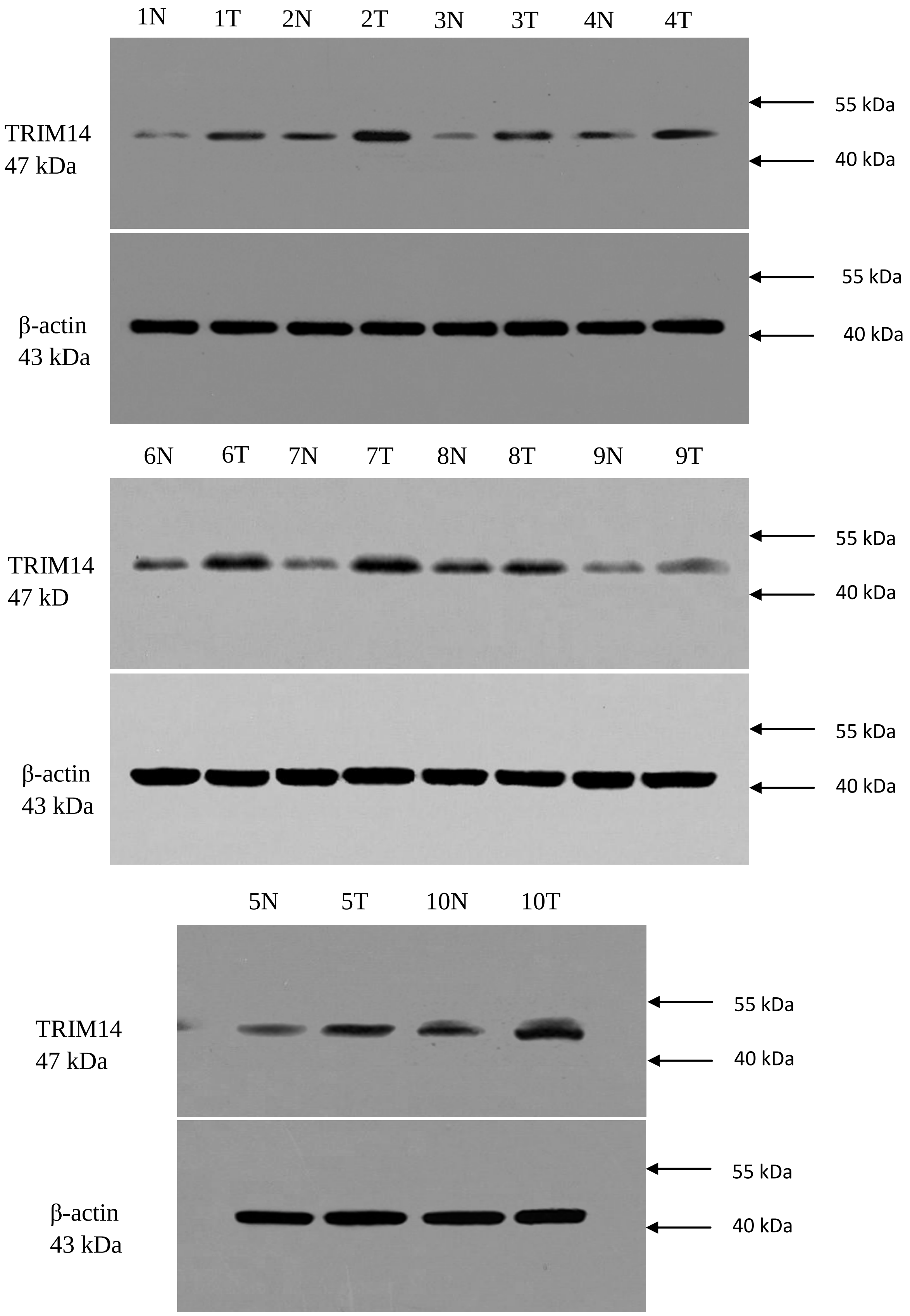
1N 1T 2N 2T 3N 3T 4N 4T
TRIM14
47 kDa
55 kDa
40 kDa
β-actin
43 kDa
55 kDa
40 kDa
6N 6T 7N 7T 8N 8T 9N 9T
TRIM14
47 kD
55 kDa
40 kDa
β-actin
43 kDa
55 kDa
40 kDa
5N 5T 10N 10T
TRIM14
47 kDa
55 kDa
40 kDa
β-actin
43 kDa
55 kDa
40 kDa

Figure 3A and 4A

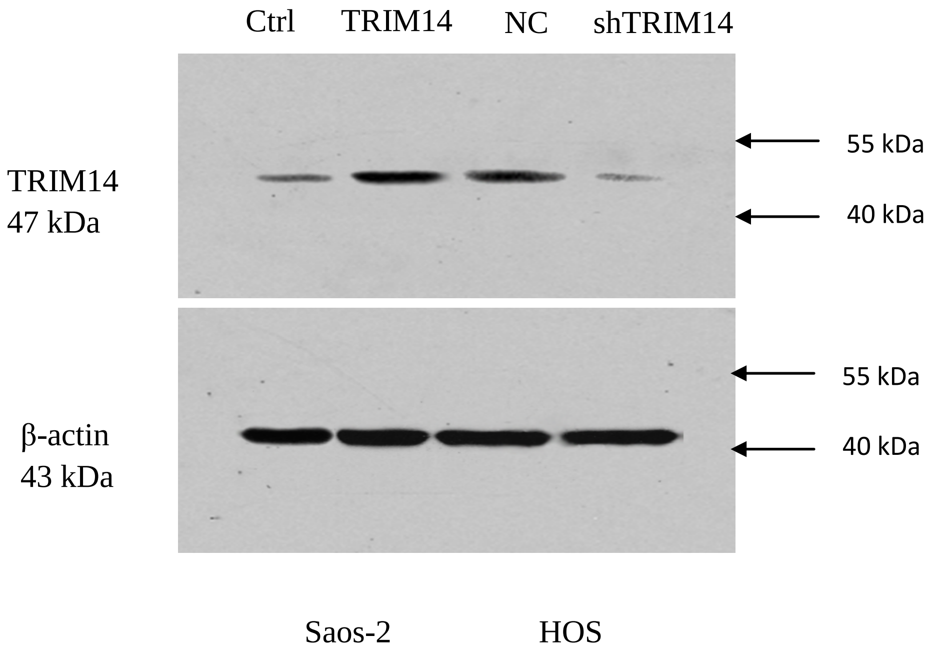

Figure 3F and 4F

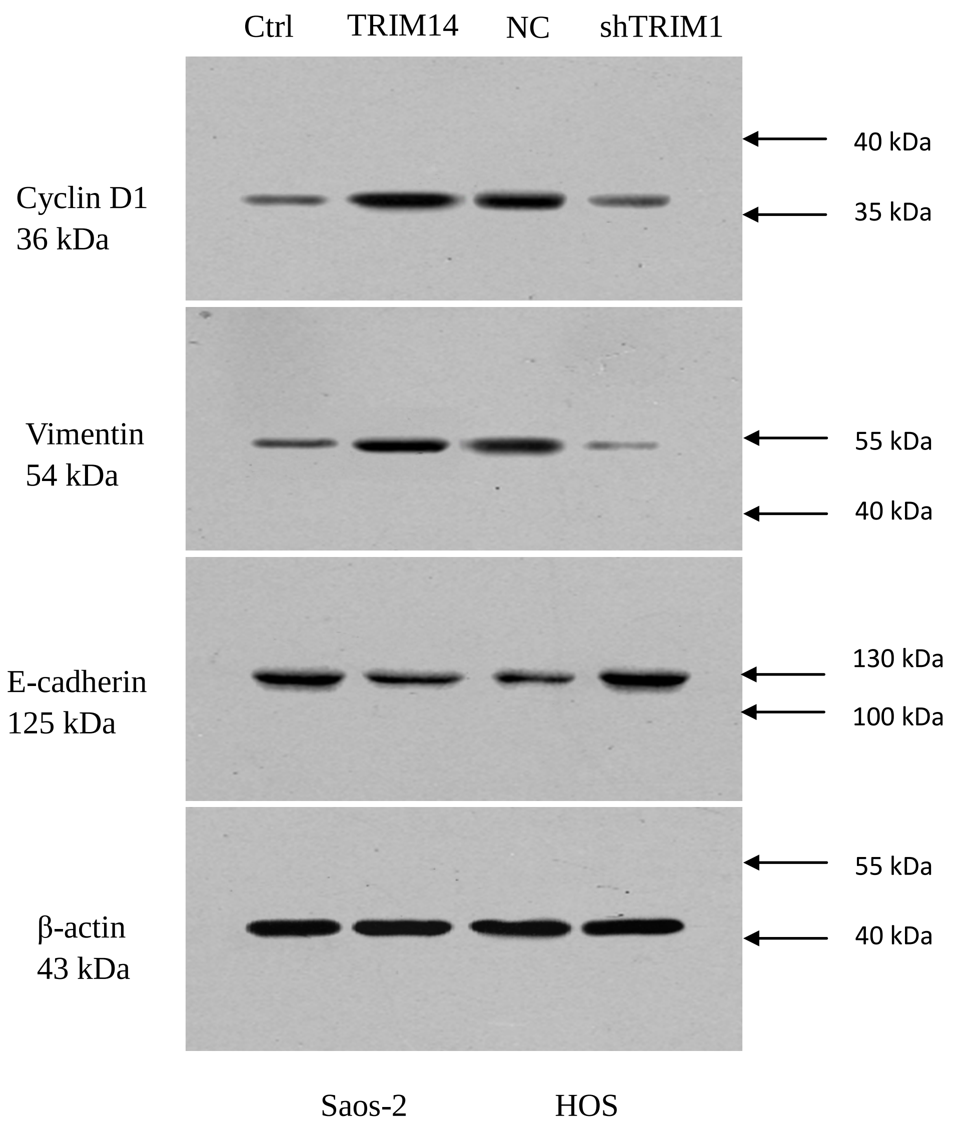

Figure 6A

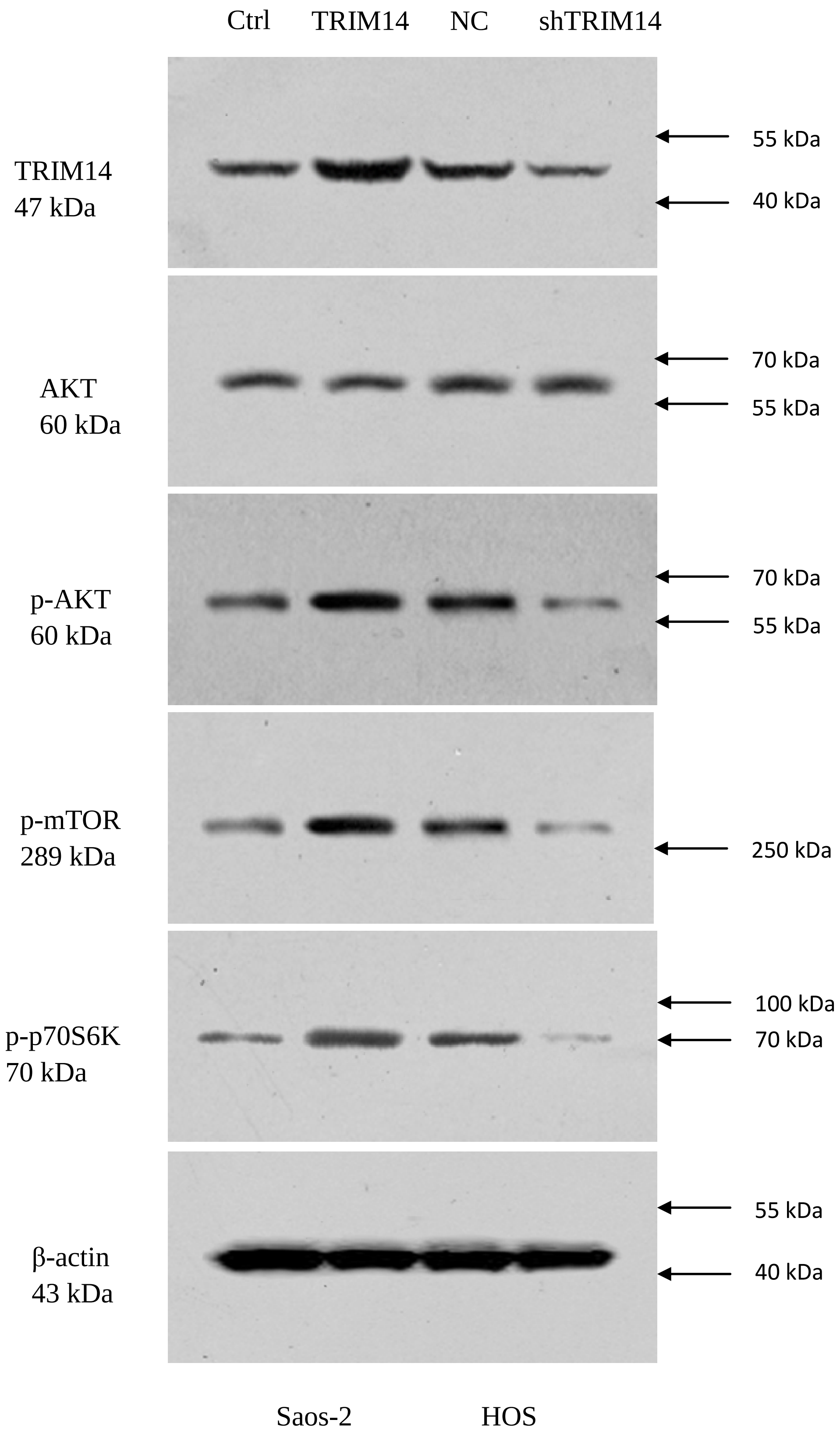
Ctrl
TRIM14
NC
shTRIM14
TRIM14
47 kDa
55 kDa
40 kDa
AKT
60 kDa
70 kDa
55 kDa
p-AKT
60 kDa
70 kDa
55 kDa
p-mTOR
289 kDa
250 kDa
p-p70S6K
70 kDa
100 kDa
70 kDa
β-actin
43 kDa
55 kDa
40 kDa
Saos-2
HOS

Figure 6D

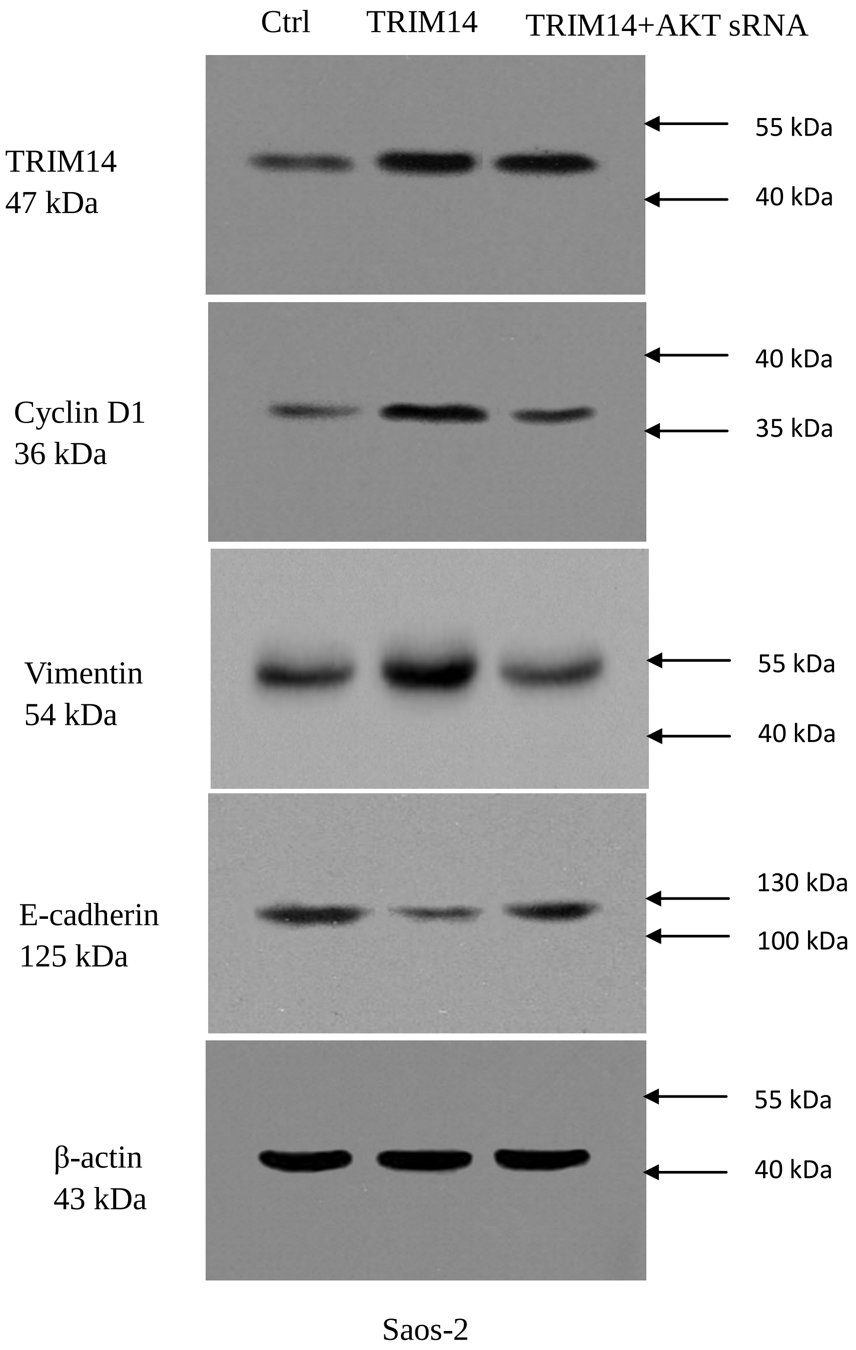
Ctrl
TRIM14
TRIM14+AKT sRNA
TRIM14
47 kDa
55 kDa
40 kDa
Cyclin D1
36 kDa
40 kDa
35 kDa
Vimentin
54 kDa
55 kDa
40 kDa
E-cadherin
125 kDa
130 kDa
100 kDa
β-actin
43 kDa
55 kDa
40 kDa
Saos-2

Supplementary Fig 1A

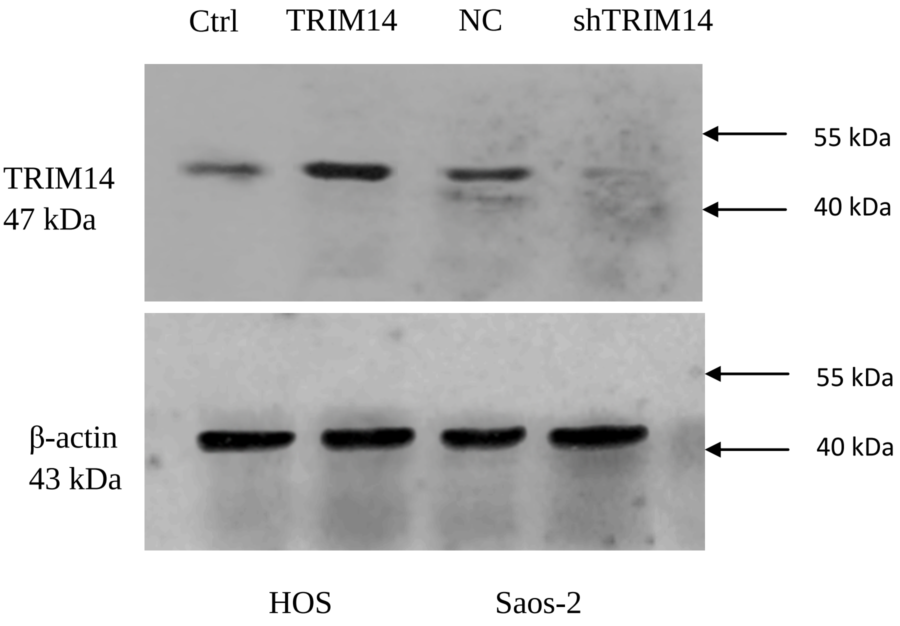

Supplementary Fig 1F

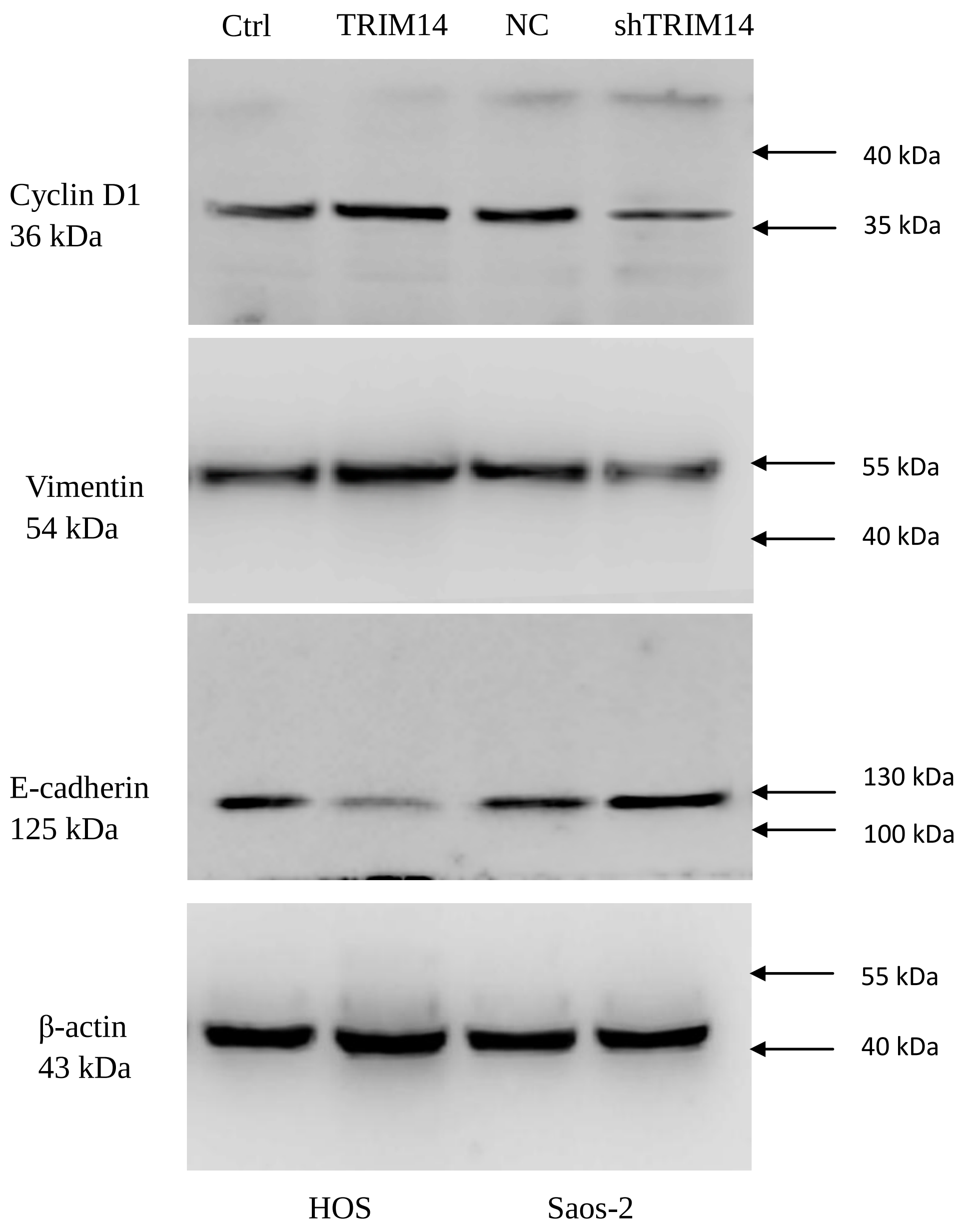

Supplementary Fig 2

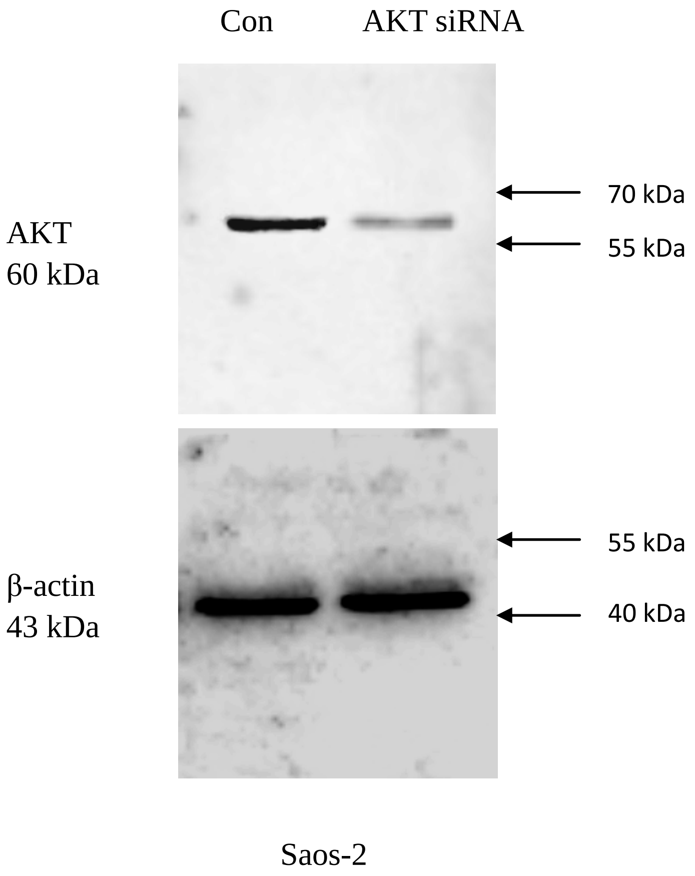

Supplement: Supplementary Data [file srep42411-s1.pdf]
